# Electromagnetic Interference in Implantable Rhythm Devices - The Indian Scenario

**Published:** 2002-07-01

**Authors:** Johnson Francis

**Affiliations:** Associate Professor of Cardiology, Medical College Calicut, Kerala, India

Implantable rhythm device (IRD) is the generic name for the group of implantable devices used for diagnosis and treatment of cardiac arrhythmias. Devices in this category include cardiac pacemakers, implantable cardioverter defibrillators and implantable loop recorders. Since these devices have complex microelectronic circuitry and use electromagnetic waves for communication, they are susceptible to interference from extraneous sources of electromagnetic radiation and magnetic energy. Electromagnetic interference (EMI) is generally not a major problem outside of the hospital environment. The most important interactions occur when a patient is subjected to medical procedures such as magnetic resonance imaging (MRI), electrocautery and radiation therapy. Two articles in this issue of the journal discusses various aspects of  EMI on IRD [1,2]. Together these articles provide a good review of the various sources of EMI and their interaction with IRD for the treating physician.

Prof. Seymour Furman has given an excellent suggestion during his editorial preview of these articles. He has called upon the Indian investigators to assess the situation in the local community. Shree Pacetronix Ltd., the only Indian manufacturer of pacemakers, has informed that no case of EMI has been reported in their pacemakers, though a well designed study to test this aspect has not been conducted so far. They also mention that their pacemakers incorporate EMI protection. Largest number of devices implanted in the country are from Medtronic Inc. They have not received reports of EMI from India. It is likely that most or all instances of EMI may have gone unreported.

Cell phones are fast becoming the common man's commodity in Indian cities and it is likely to be the single most common source for EMI. Cell phone operators in India follow the European standards of GSM (Global System Mobile) cellular telephony.  D-net (900 MHz, digital pulsed) and the E-net (1,800 MHz, digital pulsed) are being used by operators in the country. C-net (450 MHz, analogue) which was being used in European countries, is not being used by any of the major operators in the country. An IRD is most vulnerable to EMI during interrogation and programming. It is not advisable to have an activated cell phone in the vicinity during this process. Pacemaker patients really suffering from EMI due to mobile phones are very rare unless the phone is  positioned in the pocket just over the pulse generator. The contralateral pocket or the belt position of the mobile phone guarantees undisturbed operation of the pacemaker in most patients. A risk analysis reveals that the portion of patients really suffering from EMI due to mobile phones is about 1 out of 100,000 [3]. As Prof. Furman suggests, keep a cell phone in the opposite hand and ear from the implanted device and enjoy life.

MRI is an important diagnostic modality in various disciplines of medicine. A number of patients are put through MRI for orthopedic purposes without recognition of their IRD. In general, MRI is contraindicated for a patient with an IRD; but recent studies have shown that MRI using 0.5 Tesla is unlikely to produce irreversible changes in the pacemaker [4]. If a patient with an IRD badly needs an MRI, it would be worthwhile to check up whether the local system is operating at 0.5 Tesla or above. Higher magnetic fields give faster acquisition of images, but are more likely to interfere with the function of the IRD. Systems working at 0.5 Tesla are available in the country so that it may be considered in such situations. Interrogating and reprogramming the IRD before and after an unavoidable MRI is mandatory.

According to a press report which appeared in February 2002, Wilson Greatbatch, pacemaker pioneer, was planning to build an MRI-proof pacemaker with fiber optic leads and low-power semiconductor laser [5]. If his efforts bear fruit, in future we may be able to subject patients with an implanted pacemaker to MRI without any hesitation.

Radiation therapy with the IRD in the radiation field is likely to destroy the device by damaging the very sensitive CMOS (Complementary Metal Oxide Semiconductor) circuitry. In case of dire necessity, it is advisable to explant the pacemaker and reimplant it in a different location. Patients with IRD are advised not to linger within weapons detectors or theft detectors. The potential for exposure to such devices are rapidly on the increase in our country due to various reasons.

An increasing number of pacemaker-bearing individuals are being transported by air. Although modern pacemakers are effectively shielded from electromagnetic interference (EMI), the magnitude of electromagnetic radiation in cockpits of general aviation aircraft is higher and of a different nature than experienced in daily life. A recent study showed that modern pacemakers are unaffected by EMI in the cockpit environment of single-engine fixed-wing aircraft [6].

In conclusion, most patients with IRD can lead a near normal life with certain precautions to avoid EMI. It is important that the treating physician be aware of the potential sources of EMI to which each particular individual is likely to be exposed and choose the type of device and site of implant accordingly.
